# Costs and cost drivers of comprehensive sexual reproductive health services to female sex workers in Kenya

**DOI:** 10.1186/s12913-024-11293-5

**Published:** 2024-07-17

**Authors:** Griffins O. Manguro, Urbanus Mutuku Kioko, Gerald Githinji, Patricia Owira, Lillian Langat, Dan Okoro, Marleen Temmerman, Stanley Luchters

**Affiliations:** 1 Department of Public Health and Primary Care, Faculty of Medicine and Health Sciences, Ghent University, Ghent, Belgium; 2 University of Nairobi School of Economics, Nairobi, Kenya; 3 Monitoring and Evaluation, International Centre for Reproductive Health Kenya, Mombasa, Kenya; 4 UNFPA Kenya Office, Nairobi, Kenya; 5 Aga Khan University Centre for Excellence in Women and Child Health, Nairobi, Kenya; 6 Liverpool School of Tropical Medicine (LSTM), Liverpool, UK; 7 Centre for Sexual Health and, HIV/AIDS Research (CeSHHAR), Harare, Zimbabwe

**Keywords:** Cost, Female sex workers, HIV program, Kenya

## Abstract

**Background:**

Comprehensive sexual reproductive health (SRH) programs for female sex workers (FSW) offering clinical, behavioural, and structural interventions have contributed to declining rates of HIV in this population. However, data on costs and cost drivers is needed to support programs and their donors to better allocate resources, make an investment case for continued funding, and to identify areas of improvement in program design and implementation. We aimed to estimate the annual per-FSW costs of comprehensive services for a standalone FSW program in Kenya.

**Methods:**

We implemented a top–bottom and activity-based costing study of comprehensive FSW services at two drop-in centres (DICs), Mtwapa and Kilifi town, in Kilifi County, Kenya. Service costs were obtained from routinely collected patient data during FSW scheduled and unscheduled visits using Kenyan Ministry of Health records. Costing data were from the program and organization’s expenditure reports, cross checked against bank documents and supported by information from in-depth interviews. Data were collected retrospectively for the fiscal year 2019. We obtained approval from the AMREF Research Ethics Committee (AMREF-ESRC P862/2020).

**Results:**

In 2019, the unit cost of comprehensive services was 105.93 USD per FSW per year, roughly equivalent to 10,593 Kenya shillings. Costs were higher at Mtwapa DICs compared to Kilifi town DIC; 121.90 USD and 89.90 USD respectively. HIV counselling and testing cost 63.90 USD per person, PrEP was 34.20 USD and family planning was 9.93 USD. Of the total costs, staff salaries accounted for about 60%. Adjusted for inflation, costs in 2024 would be approximately 146.60.

**Conclusion:**

Programs should strive to maximize the number of FSW served to benefit from economies of scale. Given that personnel costs contribute most to the unit costs, programs should consider alternative designs which reduce personnel and other costs.

**Supplementary Information:**

The online version contains supplementary material available at 10.1186/s12913-024-11293-5.

## Introduction

In the last decade, many sub-Saharan African (SSA) countries have reported marked progress in reducing new HIV infections, increasing access to antiretroviral treatment (ART), and reducing AIDS-related deaths. Between 2010 and 2015, the Eastern and Central African region reported reductions of 14% and 38% in new HIV infections and AIDS-related deaths respectively, indicating noteworthy progress in the HIV response [1]. Such progress is attributed to many things including successful prevention programs, early initiation and expanded access to ART, oral pre-exposure prophylaxis (PrEP), and successful programs that target key populations (KP) [2–5].

In Kenya, key populations (KP) which include female and male sex workers (FSW/MSW), men who have sex with men (MSM), people who inject drugs (PWID), and transgender persons currently make up about one third of newly infected persons [6]. Additionally, FSW also contribute significantly to new infections through their general population sexual partners [7]. Fortunately, many African countries are reporting declining HIV prevalence among FSW. Our 2016 study on sex workers in Mombasa, Kenya, reported a prevalence of 12%, almost half of what was reported in previous national estimates, and modelling showed that prevalence had declined by 30% between 1993 and 2016 [8, 9]. Although it is likely that these declines reflect and are linked to similar trends in the general population, FSW-targeted programs have no doubt played a significant role [10].

Most KP programs in Kenya are donor funded. By 2021, there were about 100 such programs run by non-governmental organizations (NGOs) and KP community-led groups, serving about 207,000 FSW, 51,000 MSM, and 6,000 transgender persons [11]. The two main sources of funding are the US President's Emergency Plan for AIDS Relief (PEPFAR), and the Global Fund for AIDS, Tuberculosis, and Malaria [11]. The Kenyan National AIDS and STI Control Program (NASCOP) develops guidelines, monitors and evaluates quality and outcomes, and coordinates funding at the national level [11]. Although KP programs have always offered HIV prevention and treatment services as well as structural interventions, recent approaches by PEPFAR focus on HIV case identification and linkage to care [11]. This shift from a comprehensive community-led program offering a balance of biomedical, behavioural and structural interventions to one focused on clinical services and clinical outcomes could undermine the gains made over the years by eliminating activities that get KP interested and engaged in programs [12] and proven to have a significant impact in reducing the risk of HIV. The shift is mostly due to reductions in overall global funding for HIV and the need to double down on successful interventions [12].

A significant challenge in securing funding for FSW programs in African countries is the lack of accurate data on the per-person costs of delivering comprehensive services or the cost-effectiveness of HIV programs targeting FSW [11, 13]. By comprehensive services, we mean biomedical, behavioural, and structural interventions. Several studies have attempted to estimate these costs to inform FSW program budgets. However, these studies mostly focus on costing HIV care services alone, such as HIV testing, antiretroviral treatment (ART), and oral PrEP and largely overlook other clinical services (such as contraceptives, mental health care, and interventions for alcohol and drug use) behavioural and structural interventions (for example peer education, mapping sex worker locations, and sexual and gender-based violence interventions) [14]. Because of this lack of completeness FSW implementors, national programs, and donors often rely on incomplete data when making budgets, leading to under-resourced programs.

This study was conducted with the aim of: Estimating the per-person cost of comprehensive services in an ongoing FSW program in Kenya providing a mix of behavioural, biomedical and structural interventions; estimating the cost of specific clinical services (HIV testing, family planning, and oral PrEP), and estimating the contribution of behavioural and structural interventions to the overall per-person costs. By generating such comprehensive cost data, this study will provide FSW program implementers, national programs, and donors with the necessary data to make realistic budgets for more efficient programs. Additionally, implementers and national programs will have more accurate and complete estimates to use when negotiating with donors.

## Materials and methods

### Program description

Since 2014, the International Centre for Reproductive Health Kenya (ICRHK), a Kenyan research NGO, has implemented a program targeting to improve access to comprehensive sexual and reproductive health services to FSW in two towns in Kilifi County, Kenya. This program is funded by the United Nations Population Fund (UNFPA). ICRHK has extensive experience conducting research and interventions for FSWs in Kenya and has significantly contributed to building evidence to the national policies and guidelines [8, 15–19].

### Program design

The program follows the national (NASCOP) guidelines for FSW HIV and STI programming which recommend a peer-led approach and a balanced mix of behavioural, biomedical, and structural interventions [20]. The evidence behind adding behavioural and structural interventions, in addition to biomedical HIV prevention interventions to reduce HIV in FSW and its effectiveness has been published before. In the peer-led approach, peer educators (PEs) are engaged to educate fellow FSWs, promote and distribute condoms, and refer them for clinical services [15, 20]. Biomedical services such as HTS, PrEP and ART, and behavioural interventions such as counselling for alcohol addiction are provided through special clinics, drop-in centres (DICs), that are established and run by the program. Structural interventions, which aim to make the environment within which FSW operate, for example holding meetings to engage hotspot owners and the police to prevent and respond to sexual violence are also supported by the program. These have been shown to be effective in reducing FSW risk of infection [20]. The two ICRHK DICs in Kilifi County are in Mtwapa and Kilifi Townships, which are about an hour apart by car. The DIC in Mtwapa was the first in the region and was established in 2013. The DIC in Kilifi town is smaller, opened in 2017 and offered HIV testing as the only clinical service until 2019. Mtwapa is a cosmopolitan town, with a population of approximately 100,000. Kilifi town is smaller than Mtwapa and is mostly rural with an estimated population of 60,000.

The peer-led approach and the evidence of its effectiveness in FSW programs has been described in various publications [15, 20, 21]. In the ICRHK program, each peer educator was assigned a cohort of 60 to 80 FSWs peers who they contacted at least once a month. They were supervised by Outreach Workers (OW), and each OW supervised 15 peer educators as per the guidelines [20]. Peer educators underwent a five-day training using a NASCOP curriculum at the time of recruitment, and each year, they underwent a one-day refresher training. Each peer educator received a monthly stipend of approximately 35 USD. Outreach Workers received 75 USD and a further 20 USD (total 95 USD) per month to cover transportation costs. Peer educators and Outreach Workers also received 5 USD transport reimbursement during each outreach. All these were part of the program costs.

For clinical services including HIV testing and STI screening, FSWs were scheduled for visits to the DICs once every quarter. HIV positive FSW on ART were also required to visit the DIC every three months for medication refill, and for clinical and immunological monitoring once every year. At each DIC, two clinical officers were employed full-time. Additional clinicians were engaged to support the full-time clinicians during outreach. The additional clinicians were either nurses or trained HIV testing counsellors. The clinical services offered at both DICs are outlined in Table 1. The program leased space for DICs. The location was selected by the FSWs themselves, and was required to be safe, accessible even during evening hours, and close to sex work venues. The program obtained a licence for the DICs to operate as private clinics from the Kenya Medical Practitioners and Dentists Council. HIV test kits, ART (including PrEP), family planning commodities, and medications for tuberculosis and STIs were supplied to the DICs by the Kenya Medical Supplies Authority, in the same manner as they were provided to public facilities across Kenya. These services were offered free of charge to FSW at the DICs. For FSWs living with HIV, laboratory testing for ART monitoring and for TB diagnosis was integrated into the county’s HIV and TB program which was supported by PEPFAR throughout Kenya and offered free of charge. Samples were collected at the DICs and sent to the nearby government health facility and results transmitted online.

**Table 1:** Clinical services provided to female sex workers at Mtwapa and Kilifi town drop-in centres

Clinical service	Detail on the service
HIV counselling and testing Services	• Quarterly risk assessment • Risk reduction counselling • pre-test and post-test counselling, HIV testing
Screening and management of sexually transmitted infections	• Quarterly screening for STIs through genital examination • Syndromic treatment for STIs • Counselling on STIs, Condom promotion and distribution
ART and PrEP services	• ART for all eligible individuals within two weeks of testing HIV positive • Screening and management of opportunistic infections • Positive Health, Dignity and Prevention, • Provision of oral PrEP • Testing for CD4, viral load monitoring, and resistance testing
Family Planning	• Counselling for FP use and on dual protection • Provision of FP methods (both male and female condoms, combined oral contraceptives Medroxyprogesterone acetate (DMPA), intrauterine devices, FP implants)
Screening for cervical cancer and referral	• Visual inspection under acetic acid and Lugol’s Iodine • Referral for treatment for advanced lesions
Screening and Referral for Tuberculosis	• Screening for TB for all HIV positive FSW and referral for treatment, if necessary
Screening and treatment for alcohol use disorders	• Screening for alcohol use disorders for FSW, referral for counselling
Services for sexual and gender-based violence	• SGBV response through a network of peer educators • Sensitization to identify and respond to SGBV • Management of clinical conditions from SGBV • Post-SGBV counselling • Referral to the police, referral to the SGBV Centre at the Coast Teaching and Referral Hospital • Legal follow-up of SGBV through paralegals engaged by the program

### Data sources

We costed services for the year 2019. For peer educator and clinical service data, we used quantitative data collected routinely through Ministry of Health (MOH)/NASCOP forms. Peer education data was obtained from paper-based forms completed by peer educators each time they encountered FSWs and provided outreach services. Such data included data on the number of condoms and other services FSW received. Clinical data was obtained during enrolment, follow-up routine clinic visits, and unscheduled (sick) visits. During each visit, paper-based clinical forms were completed by clinical officers and subsequently entered into an electronic database in Microsoft Access by a data assistant stationed at each DIC. The same information was used to generate quarterly program reports. To ensure completeness and correctness of the data, quarterly data quality assessments were conducted by the program.

### Data analysis

To obtain cost data, program expenditure reports and other expenditure reports from the ICRHK finance department were used. These were cross-checked against bank documents to support the expenditure. The data collectors also conducted interviews with the Finance Manager and Senior Accountant, and with project staff to provide additional information for costing. The interview guide is provided in the supplementary materials.

We summarized the social, demographic, health, and sex work characteristics of all sex workers in the program for each DIC. This data was not used for the costing but is presented to describe the social and demographic characteristics of the FSW group. The mean and standard deviation (SD) of continuous data were presented. Numbers and proportions were used to represent binary or categorical data. To compare categorical and continuous variables between the two DICs, we used a chi-squared test and a T test, respectively.

We used a top–bottom and step-down approach for costing, whereby expenditures and economic costs were allocated to the program, then to each DIC and finally to the various program activities [22, 23]. The availability of comprehensive expenditure data for both the program and the organization, time sheets, and staff for interviews made it possible to use this approach. The personnel costs section included the salaries for staff working full-time on the program such as the Project Manager, Clinical Officers, Community Mobilizer and Data Assistants, as well as salaries for staff who only provided limited time to the project such as the Country Director and Finance Manager. The latter’s costs were prorated based on time allocated to the project as per the time sheets and supported by data from the interviews. Peer educator and outreach workers’ stipends and allowances were not included in the salaries costs but were under Peer Educator Stipends, a separate program cost. Rent and utilities’ costs for the DICs were cost under rent and utilities, an independent cost activity, while the costs for ICRHK main offices and utilities were included in Administration costs. The costs associated with program monitoring and evaluation, quality improvement, and reporting were distributed across various activities. For instance, transport for data officers to the DICs for data checks during monitoring and evaluation were categorized under transport and communications. Meeting costs during data reviews were allocated to meetings. Training costs for health workers and peer educators, aimed at enhancing service quality or updating them on revised guidelines as part of quality assurance, were allocated to training. Table 2 below details the cost categories and specific activities included in each category.

**Table 2:** Cost categories and program activities included in each category

Cost category	Program activities included
Administration costs	• Primarily the Organization’s cost of supporting the program • Included rent for ICRHK main offices, utilities for ICRHK offices, and a proportion of the organization’s overall transport, communication and internet costs apportioned to the program
Personnel salaries	• The salaries of full-time staff employed in the project (Project Manager, Project Monitoring and Evaluation Officer, Data Officer, four Drop-In Center Clinicians, one Community Mobilizer, and two Data Assistants) • The salaries of other staff who provided input in the project including from management, finance and human resources. These were prorated based on their monthly timesheets
Peer educator stipend	• Peer educator and outreach worker monthly stipends
Outreach	• Cost of conducting outreaches • Included fees for renting rooms for clinical services, fees for renting a public address system to inform FSW of the outreaches (when required) • Transportation costs for clinical equipment from DICs to outreach venues • Allowances for the additional clinical officers and HTS counsellors engaged during outreach • Transport reimbursements for PE and outreach workers • Refreshment for FSW
Meetings	• Expenses related to holding meetings – e.g. meetings to review data for monitoring and evaluation, performance review meetings, meeting with the police, hotspot owners and other key stakeholders as part of structural interventions • Specific costs here included the cost of renting the meeting venue, meals and/or refreshments, stationery, and occasionally providing transport reimbursements
DIC Rent & utilities	• Costs related to running the DICs and for repairs to the DICs • They included rent, electricity, water, and repairs
Supplies	• Clinical and non-clinical supplies • Clinical costs were primarily supplies for cervical cancer screening • Nonclinical costs included cleaning supplies and stationery
Training	• PE and outreach worker training and annual refresher training • Clinician training and refresher training • Training on monitoring and evaluation
Transport and communication	• Transport and communication for program-specific activities. Includes mobile phone and internet costs for the DICs

Given that the study was carried out in 2019, we adjusted the cost of services in 2019, for inflation between 2019 and 2024 in order to obtain the approximate cost in 2024. Inflation rates were: 2020, 5.4%; 2021, 6.1%; 2022, 7.7%; 2023, 7.0%; 2023, 5.1; 2024, 5.1% (based on preliminary data from early 2024). The average annual inflation rate during this period was 6.8%. The adjusted rate was calculated using the formula:

$${\text{Cost in }2024=\text{ cost in }2019\text{ X }(1 +\text{ average annual inflation})}^{\text{number of years}}$$

The cost of HIV test and STI screening kits, ART (including PrEP), family planning (FP) commodities, and laboratory monitoring for HIV and TB were not included. There were several reasons for this: 1) The Kenya Medical Supplies Authority procured HIV and STI test kits, ART, and FP commodities and distributed these to health facilities; patients in public facilities and DICs were not charged 2) Afya Pwani, a PEPFAR-funded project coordinated sample pick-up, testing and ensured individual patient results were available through a central, online portal for all HIV and TB patients in the region at no cost to the patients; 3) The exact prices of these commodities were unavailable, and if we were to use global market prices, our estimates would have been exaggerated as governments purchase in bulk at lower prices; 4) Because these costs were already covered by other national programs and were not routinely included in FSW program budgets or scope, by including them, our estimates of FSW program costs would be exaggerated. We did include the costs of cervical cancer screening through visual inspection using acetic acid and Lugol’s Iodine because this was offered annually to all sex workers but was not covered by the KEMSA supplies and were included in the project’s budget.

The financial costing process adhered to the principles outlined in the Global Health Cost Consortium Reference Case (GHCC) [24]. The total cost of the program for the year was calculated by adding all the actual costs incurred. The annual per-FSW cost was calculated by dividing the total program cost to the weighted total number of FSW who received services during that time period. The weighting was determined by the number of visits made by each FSW.

### Ethical approval

The study obtained approval from the Amref Research Ethics Committee (AMREF-ESRC P862/2020) in Nairobi, Kenya to analyse retrospectively collected program data, costing data from program expenditure, and to conduct interviews with ICRHK staff to get more information on budgeting and cost allocation. Individual FSW data were collected during routine service delivery, so informed consent was not obtained. For ICRHK finance, administration, and program staff who answered interview questions, written informed consent was obtained. All methods were carried out in accordance with relevant guidelines and regulations pertaining to research in human subjects.

## Results

### Summary of characteristics of services and of FSWs

Between January and December 2019, 1,964 FSW received comprehensive services at the two DICs. Of these, 1,175 were served in Mtwapa and 789 in Kilifi town. There were 4,358 visits in Mtwapa and 1,968 in Kilifi town, an average of 3.7 (Standard deviation [SD] 2.9) and 2.4 (SD 1.8) visits per FSW per year in Mtwapa and Kilifi town respectively. Visits lasted between 15 and 45 min, with an average of 28 min per visit. Fifteen peer educators were engaged to cover sex work venues in Mtwapa and 12 in Kilifi town. In Mtwapa, each PE maintained a cohort of 79 FSW while in Kilifi town, each had a cohort of 65.3.

In brief, the majority of the FSW were aged between 21 and 30 (63.1%; Table 1)) with a median age of 27 years. HIV prevalence in the total FSW population was 6.3%. There were significant differences in social and demographic characteristics (age, education, marital status), clinical characteristics (HIV and STI prevalence) and sexual characteristics between those served in Mtwapa and Kilifi DICs. 

### Cost of FSW services

**Fig. 1:**
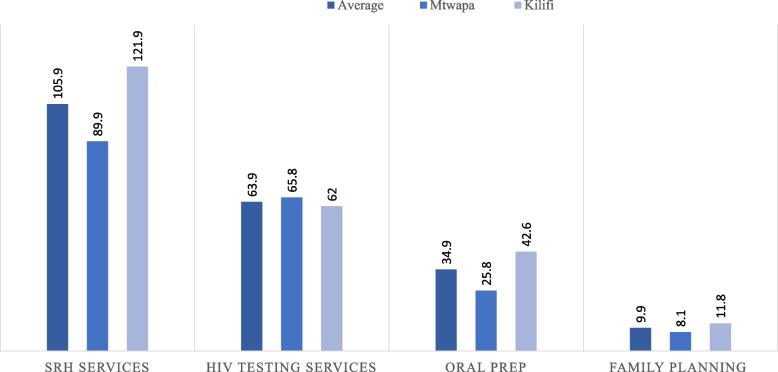
Unit cost of FSW services in US Dollars. A bar chart representing the cost in US dollars for 1) all SRH services, 2) HIV testing services, 3) Oral Pre exposure prophylaxis services, and 4) family planning services for an individual FSW for one year for the program, and at Mtwapa and Kilifi town DICs

In 2019, the cost of providing HIV and other services to one FSW for the year was 105.93 USD, which is roughly equivalent to 10,593 Kenyan Shillings (Kshs) based on the 2019 exchange rate of 1 USD = 100 Kshs. The annual cost per FSW in Mtwapa was 89.90 USD, and in Kilifi it was 121.90 USD. The cost of HIV testing services (HTS) was 63.90 USD per FSW per year across the program, 65.80 USD in Mtwapa, and 62.00 USD in Kilifi DIC. For oral PrEP services, the cost was 34.20 USD per FSW per year in the overall program, 25.80 USD in Mtwapa and 42.60 USD in Kilifi DIC. FP services cost 9.93 USD across the program, 8.10 USD in Mtwapa and 11.80 USD in Kilifi. Figure 1 presents the cost of services across the program and at the two DICs. Adjusted for inflation between 2019 and 2024, the estimated cost of services per FSW in 2024 would be USD 146.50. In Mtwapa, services would cost USD 120.90, while in Kilifi DIC, they would be USD 162.62. For the individual activities of the program, the adjusted costs for 2024 would be USD 85.90 for HTS, USD 45.98 for PrEP, and USD 13.35 for FP.

### Breakdown of financial costs for different activities

**Fig. 2:**
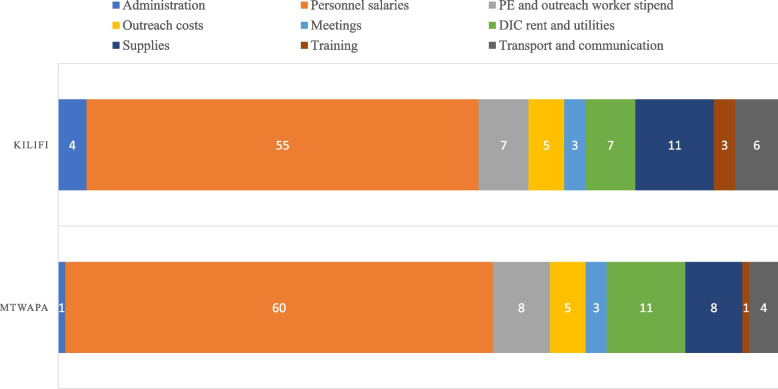
Key program activities and the proportion (in percentages) they contribute to the unit cost. A colour-coded horizontal stacked bar chart depicting key program activities and the proportion (percentage) they contribute to the per-year unit cost of FSW services at Mtwapa and Kilifi Town DICs

When we looked at how various program activities contributed to the overall costs, personnel costs were the greatest contributor. In Mtwapa, personnel costs accounted for about 60% of the overall costs (USD 53.94) and in Kilifi, it was 55% (USD 67.05). Other significant cost drivers were rent and utilities for the DICs, (11%, USD 9.90 in Mtwapa and 8%, USD 9.75 in Kilifi), PE/Outreach Worker stipend (8%, USD 7.19 in Mtwapa and 8% USD 9.75 in Kilifi.). Figure 2 presents the key program activities and the proportion they contributed to the overall unit costs at each DIC.

## Discussion

We carried out this study to estimate the unit cost of comprehensive FSW services in two drop-in centres in Mtwapa and Kilifi town, Kenya, funded and implemented as one program and by the same Organization. We estimated the costs of a program that provided comprehensive services using the NASCOP-recommended approach which emphasized on a peer-led implementation with an equal mix of biomedical, behavioural and structural interventions. This research builds on previous research that have demonstrated the success of HIV outcomes in FSW programs. While our previous research demonstrated that FSW-targeted programs as designed effectively reduced HIV incidence and prevalence, our goal here was to provide national programs and implementing partners with an estimate of the per-person cost of service delivery to guide funding allocation and to identify cost drivers. We found that the cost of services was 105.93 USD per FSW per year on average, with personnel accounting for nearly two-thirds of the cost. Furthermore, service costs were lower at the Mtwapa drop-in centre (DIC), compared to Kilifi DIC.

When we compared our unit costs to other studies, we found large variations, ranging from as little as 10.70 USD in India to 1098.00 USD in Burkina Faso [25–27]. This is not surprising; multiple factors contribute to the per-person costs; FSW programs have complex and different designs, different studies include different costs and studies perform analyses differently. The FSW population reached, services provided, in-country cost of goods, maturity of the program and the organization-level efficiency of the implementing partner all contribute to the per-person costs [25–32]. Large programs that serve many FSW benefit from economies of scale which has been shown to result in lower unit costs even when the projects are implemented by the same organization [27, 31]. FSW programs that offer comprehensive antiretroviral treatment are more expensive than programs that offer peer education, counselling and testing alone because of the additional service delivery costs. Structural interventions, such as advocacy to decriminalize sex work, and measures to address violence when added to FSW interventions also increase the unit cost of programs [28]. Community-based organizations, which generally have simpler structures and where salaries for management and other staff are likely lower are also likely to have cheaper running costs than local or international NGOs [26]. Recently established programs have also been reported to cost more, presumably due to higher administrative costs associated with more intense supervision and coordination, and project overhead costs for FSW projects are likely to reduce over time [25]. In Burkina Faso, for example, Cianci et al. reported an annual cost of 1098.00 USD per FSW, nearly ten times what we report [25]. Included in their cost analyses were ART, laboratory testing for HIV monitoring (CD4 and viral load), and treatment for opportunistic infections, which we did not. Their clinic also served 305 FSW per year, roughly one-fifth of our population, and approximately 60% of their FSW population were HIV positive on ART, including second-line ART. Conversely, in Nigeria, Nance et al. sampled 31 community-based organizations and reported a mean cost of services of 22 USD per FSW per year [27]. Only HIV education, counselling and testing with referral to other facilities were provided. It is worth noting that in our study, the unit cost at Mtwapa DIC was lower than at Kilifi town DIC, much as both DICs were operated by the same ICRHK program which underscores the importance of economies of scale as a cost determinant. This was also reported by the Avahan Program in India [25].

That Personnel costs contributed most to the per-person cost is consistent not only with FSW studies, but with other HIV service programs such as Prevention of Mother-to-Child Transmission (PMTCT), Voluntary Male Medical Circumcision (VMMC), and Pre-Exposure Prophylaxis (PrEP), and across various geographies [30, 33, 34]. Personnel costs in this study included direct service provider salaries, such as nurses and clinical officers, as well as time and effort compensation for monitoring and evaluation and project management. While we were unable to provide a breakdown of the proportions for each service level (direct service delivery personnel costs versus costs for support project staff), studies such as the Avahan study report that direct service delivery personnel costs can account for up to 65% of unit costs [25]. There are multiple ways through which FSW programs can improve their efficiency and reduce their costs for service delivery. For example, current Kenyan ART guidelines recommend differentiated service delivery for PLHIV beyond the first six months of ART for patients who are established to be adherent to medication [35] and such patients can be followed up less frequently, with up to six months between clinical appointments. This means that programs can deliver quality clinical care for more FSW with less clinical staff. The PEPFAR FY2024 technical considerations also recommend that KP programs consider a set of optimized testing approaches that includes social network strategy testing, index testing, risk network testing, self-testing, social media and information communication technology platforms to complement standard venue based HTS [36]. These approaches could mean that a greater number of FSW can receive HIV services outside the DICs.

Another approach to reduce costs and ensure sustainability, which has been recommended by NASCOP would be to integrate FSW services into the routine services in public and private health facilities instead of stand-alone DICs. Such an approach would mainstream FSW services and reduce stigma and discrimination. Jilinde, Kenya's largest PrEP scale-up program successfully piloted PrEP services for FSW in public and private health facilities [37]. Similarly, other FSW programs have established "link desks" within public health facilities, whereby a peer educator is assigned to a “link desk” to help FSW visiting the facility navigate through care. Protocols on providing FSW services are available, and these programs ensure strong links between Peer Educators and the facility to minimize referral loss and establish safe spaces for FSWs peer support. The staff at the health facility are also trained to provide non-discriminatory, stigma-free services. One big disadvantage of such an approach of integrating FSW services into available public and private health facilities is that FSWs typically prefer DIC services because of the privacy and tailored services and FSW could engage less with programs and interventions when services are integrated [38]. Additionally, it is challenging for health facilities already stretched to provide focused care to a single population. It may also counterfactually increase stigma against FSW when the receive preferential care at public and private health facilities.

It is worth noting that family planning (FP) constituted only 10% of the total unit cost. This means that FP services can be easily added without significantly driving up the unit cost. While FP can be considered a cost-effective intervention, many FSW programs do not include FP services into the programs’ design, DICs do not routinely stock FP commodities and clients are often referred elsewhere. Alternatively, FSW pay for FP services in private facilities despite the ease of availing them in public facilities [39]. One of the major gaps for FSW programs in Africa is the inability to integrate other relevant health issues into HIV services; programs have been criticized for focusing on HIV and ignoring other health issues that contribute to the overall wellbeing of FSW, even when such interventions are inexpensive and easily integrated into whatever is already in place. However, this could be interpreted as inflexibility, which is common in many donor programs; FSW programs are frequently funded by HIV-designated funds, which are frequently ineligible for use to support other health issues.

In summary, this study provides a unit cost estimate for comprehensive FSW SRH programming providing a balance of biomedical, behavioural, and structural interventions, and includes both service delivery and above service costs. FSW programs should consider using these estimates when budgeting and advocating to donors. Our estimates of cost drivers should also guide policy makers in making decisions on how to structure programs and maximize cost efficiency.

Our study has some significant limitations. First, we were unable to conduct a cost-effectiveness analysis to provide robust evidence that the program at this cost “works”. The cost-effectiveness would be a critical piece of evidence for funding justification. However, this was not our objective for this study as we had inadequate data on the program outcomes. Secondly, the cost analysis was from a single FSW program in two DICs in Kenya’s Coast region, and therefore, the findings may be considered not nationally representative. However, we believe that the programs’ design accurately represents the NASCOP model used by most programs in Kenya, and we therefore provide accurate information that can inform advocacy and decision-making at both the program and national levels. Thirdly, our cost estimates did not include the cost of HIV test kits, ART, STI medication, and laboratory tests, which lead to an underestimate. However, it is important to note that these costs are not typically included in FSW program budgets, and our estimates may accurately represent the actual costs incurred by programs. Finally, the data was from 2019, and may not accurately reflect the current cost of services. We have updated the cost based on the present inflation rates however, to provide an estimate for 2024. One major advantage of our study is that it is the first in Kenya to present a unit cost of comprehensive services provided to FSWs, whereas previous studies have only estimated the unit cost of HTS, ART, or PREP separately [28].

## Conclusion

Programs can benefit in multiple ways from understanding the unit cost of comprehensive services provided to FSWs. First, it can assist programs in advocating for increased funding or convincing funders to include the cost of essential program activities, such as structural interventions. Second, cost estimates can help programs identify the primary cost drivers and propose interventions to optimize program design. Finally, these cost estimates can serve as a guide for countries that do not yet have cost estimates.

### Supplementary Information

Supplementary Material 1.

## Data Availability

The datasets containing individual FSW service data generated and/or analysed during the current study are available from the corresponding author on reasonable request.

## Abbreviations

SSA: Sub Saharan Africa
HIV: Human Immunodeficiency Virus
ART: Antiretroviral treatment
KP: Key populations
PrEP: Pre exposure prophylaxis
MSM: Men having sex with men
PWID: People who inject drugs
FSW: Female sex workers
NGO: Non-governmental organization
PEPFAR: President’s Emergency Plan for AIDS Relief
NASCOP: National AIDS and STI Control Program
ICRHK: International Centre for Reproductive Health Kenya
UNFPA: United Nations Population Fund
STIs: Sexually Transmitted Infections
DICs: Drop-in Centres
Pes: Peer Educators
USD: US Dollars
KEMSA: Kenya Medical Supplies Authority
TB: Tuberculosis
SD: Standard Deviation
PMTCT: Prevention of Mother to child Transmission
VMMC: Voluntary Male Medical Circumcision
HTS: HIV Testing Services
FP: Family Planning
SRH: Sexual and Reproductive Health
SGBV: Sexual and gender based violence
