# Supplementary material for: Costs and cost drivers of comprehensive sexual reproductive health services to female sex workers in Kenya
Source: BMC Health Serv Res. 2024 Jul 17;24:822. doi: 10.1186/s12913-024-11293-5 (PMC11253356; doi:10.1186/s12913-024-11293-5)
Supplement: Supplementary file 1 — Supplementary Material 1. [file 12913_2024_11293_MOESM1_ESM.pdf]

International Centre for Reproductive Health Kenya; the Female Sex Worker Costing Study, Kilifi.

AMREF-ESRC P862/2020

Key Informant Interview Guide.

This is a key informant interview guide for the FSW costing study. For each participant, please fill the first section with the correct details.

Interview Date	
Time interview started	
Time interview completed	
Participant Code	
Participant Position/title	
Completed by:	

Interview should be conducted in private. If possible, and if available please request to verify information from existing and available documents.

Questions

1. What is your title?
    a. *Please share a short description of what you do in the organization and in the FSW project*.
    b. How long have you been in the organization? How long have you been engaged in the project?

2. Do you participate in preparing project budgets?
    a. What is your involvement in the budget for *this specific project*?

3. Could you explain the process by which you create the budget for this FSW project?
    a. For instance, is your budget open or does the funder place a cap on it?
    b. Does the funder have criteria for you to follow when budgeting for activities, or is it up to you and the organization?
    c. Are there any recommendations available regarding the optimal ratios for different program activities? *For instance, is there a maximum percentage for things like staff pay, transportation, etc.?*

4. Do you prepare one budget for the entire FSW project, or do you prepare budgets independently for the two drop-in centers?

   a. How often do you prepare budgets for this project? Annually? Every three months?

5. How about reports, how often do you prepare expenditure reports for this project? *Do you do any field verification to ensure that data you receive from the DICs corroborates with actual field expenditure?*

6. When making the budget, how do you allocate the cost of personnel who are not one hundred percent engaged by the project but who contribute time and effort to the project? (for example, Finance, HR staff, drivers etc.)
   a. How do you ensure that the allocated level of effort that you include in the budget is accurate and represents the time spent on the project?
   b. Does this vary over time, (*for example in Quarter 1 you'd budget a given proportion to an accountant and in the next quarter this proportion is) different?*
   c. How do you verify how much time/effort these staff spend on the project?
   d. Are there times when you have a difference between the allocated time/level of effort in the budget and the actual time spent? *For example, are there instances when you budget a certain proportion, but the staff spend more or less time on the project?*

7. How do you divide up the costs of operating the main office and other organizational expenses (rent, utilities, internet, travel/transport) to this project when creating the budget?
   a. How do you ensure that what is allocated to this project is accurate?
   b. Are there times when you have a difference between what you allocated and what you spent? How often does this occur?

8. For costs that are directly related to the project (for example travel and supplies) and which are incurred by both project sites at the same time, how do you split the costs between the Mtwapa and Kilifi drop-in centers?

Thank the participant for their time and willingness to participate in this key informant in-depth interview.
